# The Impact of Negative Informal Information Before a Change on Performance: A Within-Person Approach

**DOI:** 10.3390/ijerph17020670

**Published:** 2020-01-20

**Authors:** Xia Jiang, Jing Du, Jinfan Zhou, Yumeng Cui

**Affiliations:** 1 Department of Human Resources Management, Economics and Management School, Wuhan University, Wuhan 430000, China; jiangxia@whu.edu.cn (X.J.); jdu@whu.edu.cn (J.D.); 2 Department of Human Resource Management, Business School, Nanjing University, Nanjing 210093, China; 3 Human Resource Department, WISDRI Engineering & Research Incorporation Limited, Wuhan 430000, China; 28064@wisdri.com

**Keywords:** informal information, organizational change, resistance intention, within-person, performance

## Abstract

We live in a rapidly changing business environment where change has become the norm for organizations to maintain competitiveness. Although both scholars and practitioners agree that organizational change communication is important to help employees adjust to change, little is known about how negative informal information before the change affects employees’ reaction to the change and occurrence of possible within-person dynamics of resistance intention over time. Based on the construal-level theory, we used SPSS 22, AMOS 20, and HLM 6.0 as tools to explore how negative informal information affects individual performance. We used a multilevel approach to probe within-person processes among 215 MBA students in China. The results show that (1) negative informal information provided before the organizational change is positively related to the resistance intention, (2) resistance intention decreases significantly over time, and (3) negative informal information is negatively related to individual performance during the organizational change. The results from this study extend the literature on informal communication before the change and provide a dynamic perspective on the occurrence of possible within-person dynamics of resistance intention over time.

## 1. Introduction

In response to the impact of the internet, economic globalization, and various environmental factors, many organizations are finding it necessary to engage in some form of reorganization or change to sustain their success and existence [1]. Determining how to successfully undergo organizational change is a key topic for almost all organizations [2]. The cost of organizational change failures often extends beyond financial resources and time [3], affecting the success of future reform efforts and organizational culture [4]. Therefore, an effective approach is required to drive constructive change [5] and encourage employee behaviors that benefit the implementation of organizational change [2]. 

A growing consensus recognizes that organizational communication has an important impact on organizational change [6,7]. However, more attention in the change management literature has been paid to uncertainty management and organizational change communication; the majority of these studies focused on the process of change [3,6]. In reality, informal information is often circulated before the change occurs in many situations [3]. Due to the considerable impact of organizational changes, firms are often cautious about publishing any official announcement. The limited availability of formal information increases the importance of informal information as a source for employees to learn about the changes [8,9]. In other words, when organizational change communication is perceived to provide insufficient information, employees seek information through informal information or other informal channels [6]. Subsequently, poorly managed organizational change communication leads to resistance to change, magnifying the negative aspects of the change [7]. Therefore, managing and responding to informal information before the change is vital for managers.

Previous studies in the organizational sciences have not focused much on studying the dynamic trajectories within an individual [10]. Faced with negative and unfavorable information, employees may not just passively wait, but actively adjust and respond creating a problem for change management [11]. Matusik [10] explained that the reaction of employees to a change around them is partially a product of previous states. The resistance that may occur in the future depends on the past states; therefore, capturing how resistance processes change over time is crucial.

**Figure 1:**
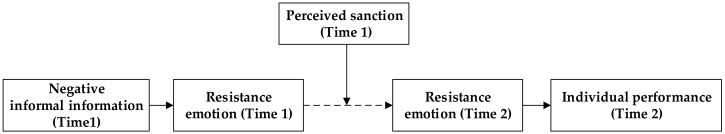
Conceptual framework.

In this study, we focused on the informal information provided before the organizational change and used a within-person approach to analyze the employees’ resistance. We aimed to identify how this process evolves and if it is possible for employees to automatically adjust to the organizational changes. To answer these questions, the remainder of this paper is structured as follows. Section 2 outlines the theoretical framework (see Figure 1) and hypotheses. The third section describes our methods. The fourth section provides data analysis and results, with the discussion in Section 5. The final section summarizes the research with an overall conclusion and outlines our contribution.

We aimed to provide upper management with a deeper understanding of the various functions of informal information within organizations. Therefore, we collected two-wave data to explore processes and changes within individuals over time, rather than examine the differences between persons as in previous between-person studies [12]. We argue that the provision of informal information is a fundamental activity and may never be completely eliminated from the organization [13]. However, it can be managed to some extent and adopted as an effective means to control individuals. Therefore, we discussed possible practical actions for upper management to encourage an optimal informal information environment.

## 2. Theory Framework and Hypotheses 

### 2.1. Negative Informal Information and Resistance Intention

Informal communication includes all types of communication that are not officially disseminated within the organization [6] such as rumor, gossip, casual conversations, urban legends, and chats [14,15]. All kinds of informal information obtained by employees before organizational change were included in our study. Informal information before change refers to an unverified piece of information about the change that is of importance to a group before the organizational change occurs [16]. Informal information is unverified and potentially useful information statements in circulation that arise in ambiguous and potentially threatening contexts and help employees to manage that threat [8]. Informal information is characterized as being emergent, spontaneous, dynamic, associative, arbitrary, interlinked, contextualized, and multi-perspective [17].

Rosnow [18] indicated four reasons for the emergence and dissemination of informal information: uncertainty of change, the importance of the change of theme, the trust of employees in information, and the anxiety of employees. Another study focused on employees’ motivation and concluded that employees disseminate informal information for four purposes: information collection and verification, social entertainment, group protection, and negative impact. Information collection and verification is the most common motivation for employees to communicate [19]. Johlke and Duhan [20] suggested that the transmission of informal information can effectively improve employees’ role cognition and reduce their resistance. Informal information dissemination is flexible and can compensate for limited formal information, so that employees can fully understand the latest developments of the company. Therefore, informal information is indispensable and plays a significant role in organizations. Smet et al. [6] suggested that lacking sufficient organizational change information increases feelings of job uncertainty and insecurity among the employees. Before the change, employees may seek information from various sources, such as leaders, colleagues, friends, or other contacts [21]. When information is insufficient or information sources are unreliable, informal communication arises due to the need for a feeling of safety [6,22]. 

Informal information before the change is an important method for employees to become aware of the uncertain change. Employees usually conduct snooping and information collection to prepare for the changes in advance. Informal information is generally divided into negative and positive information. Grosser et al. [13] studied the social network of informal information, which further affected the social influence of employees. They found that negative informal information is more likely to spread among colleagues compared to the positive information. Therefore, we speculated that negative informal information may spread faster and more widely within the organization before a change occurs in comparison with positive information. Some studies showed that changes in work patterns often place some pressure on employees, and the uncertainty about change is often greater than their positive expectations. Therefore, when employees receive negative informal information before the change, anxiety and pressure about the unknown upcoming changes arise, which triggers resistance intention. In other words, the outflow of informal information before a change increases job uncertainty and insecurity and employees are outside of their comfort zone. In our opinion, this causes discomfort and even resistance intention among the employees. Based on the above arguments, we propose the following hypothesis:

**Hypothesis** **1.** *Negative informal information is positively related to employees’ resistance intention before an organizational change occurs*.

### 2.2. Resistance Intention Decreases Over Time

Resistance intention to change is considered an attitudinal reaction of an individual’s dispositional inclination to resist change [23]. Although resistance intention may be the most direct consequence when employees become aware of organizational change, a review of the current literature suggests that research on the extent and evolution of this response over time is lacking. If employee resistance intention is temporary, the expected adjustments and psychological preparations can be implemented before the change, so that the employees can better embrace the change. However, if resistance intention persists for a long time or is converted into actions, the final effect of the change may be threatened.

According to construal level theory, when an employee first obtains informal information before an organizational change, the unknown change has a long psychological distance from the employees. Therefore, the employees’ evaluation of change is still based on the overall and simple cognition, and they believe that the change will carry them into an uncertain and unfamiliar field or violate the old interest pattern, rather than deeply analyzing the change. Therefore, since no in-depth analysis and research is conducted, it is not surprising that the first reaction is resistance.

Over time and with an in-depth understanding of relevant information, employees realize that the psychological distance is closer. Thus, employees focus more on the characteristics of the facts and details, such as thinking about the possible methods of the implementation of the change and how to manage loss after the change occurs. They constantly construct and analyze the change in their minds, eliminate uncertainty and enhance self-control. From the initial resistance to thinking about details in a later stage, employees’ psychology toward change constantly transforms over time. After obtaining informal information about the imminent change of the organization, due to the distance from the psychological comfort zone and the unknown nature of the change, this uncertainty will form resistance intention at first. However, this state may only last for a short period. Over time, employees gradually adapt to the facts and various possible consequences, and gradually begin to rethink and evaluate so that they are psychologically prepared when the change is effected [24]. 

For instance, receiving informal information about corporate personnel transfer, employees may initially react negatively to this event, but over time, they begin to pay attention to more detailed aspects and gradually think about the positive aspects to adjust their intention. Consequently, during this period, their resistance will gradually decline. Psychological adaptation and adjustment not only help them to prepare for the change in advance but also reduce the negative intention and the obstacles to the formal implementation of the change, improving the final effect. Based on the above arguments, a second hypothesis is proposed:

**Hypothesis** **2.** *The intention of resistance to organizational change decreases significantly over time*.

### 2.3. Perceived Sanction as a Contextual Facilitator of Resistance Intention

Sanction is one of the common tactics used by managers to punish employees for noncompliance [25], mainly relying upon explicit provisions or potential penalties, which makes employees feel pressure and threat from the organization, as well as forced to comply with the change. Perceived sanction refers to the perception that “I will be punished if I do not comply”. Organizational change often means that employees have to leave their psychological comfort zone so that managers need to guide employees to embrace the change and promote the implementation of change outcomes [25]. Change is usually determined by the upper levels of management, which restricts and limits employees through clear rules and regulations. For the convenience and effectiveness of management, it is impossible for leaders to have complete communication with each employee. Compared with the employees’ high-cost tactics to fully understand the meaning of change, employees perceive the threats and losses to resisting the change, which helps them to adapt to the change and achieve organizational goals more quickly. Therefore, paying attention to sanctions in organizational change can assist with understanding employees’ resistance intention.

When employees have a high perception of sanction, they recognize that they will be penalized if they do not comply with the change. Feeling pressure from the organization, employees usually have no choice but to passively accept the change or adjust their mentality, which can result in self-protection or personal gains [26]. Over time, when employees appreciate the consequences of resisting and that changing the decisions of their superiors is impossible, they usually adjust themselves and find ways to avoid punishment and ensure they smoothly weather the change period [11]. For instance, they may find reasons to convince themselves that the change is important and assume that sanction is required.

Under the perception of high levels of sanction, most employees are faced with the pressure of changing their minds or resigning. The decline rate of employees’ resistance intention will be faster over time due to the self-protection and long-term planning of employees. Conversely, under the perception of low levels of sanction, employees believe that even a boycott will not result in severe punishment. Therefore, employees may neglect or disagree with organizational changes and psychologically accept changes slowly, so employees’ resistance intention to change will decay slowly. Based on the above arguments, the following hypothesis is proposed:

**Hypothesis** **3.** *Resistance intention decreases to a greater extent when a perceived sanction is high compared to when a perceived sanction is low*.

### 2.4. Resistance Intention and Individual Performance

The resistance intention of employees during organizational change will determine their follow-up behavior and further impact their performance. Individual performance is a systematic evaluation of employees’ work behavior and outcomes and is an important evaluation indicator for employees’ salary and career development. We assume that individual performance also indirectly reflects the implementation of the change and the level of employee involvement. The resistance intention of employees indicates their attitude toward organizational change. If the resistance intention is high, employees still hold a disapproving attitude toward the change and they will not actively participate and support the change, which will affect the smooth implementation, and finally, their own work performance will decrease over time. However, if the resistance intention is low and the employees have a fair understanding of the change, they comply with it and may even help to implement the change. Therefore, we suggest that their individual performance will consequently improve. Based on the above arguments, the following hypothesis is proposed:

**Hypothesis** **4.** *Resistance intention during organizational change is negatively related to individual performance*.

## 3. Method 

### 3.1. Participants and Procedure

Following Aaker et al. [27], we adopted the recalling intention method to collect the data in two phases. During the first phase, a recruitment email was sent to MBA students at a university in central China. The candidates who fit the following requirements were selected to participate in the study: (1) employed in a full-time job, (2) in an organization that has recently experienced an organizational change with a considerable impact on the employees and (3) that has good internal communication, in which colleagues share information more frequently. These requirements were set to ensure that the participants had experienced a real organizational change situation and received informal information before the change so that they would effectively rate their subsequent reactions during the survey period. Before the formal investigation, we communicated effectively with the participants to help them fully understand the purpose and process of the survey, and emphasized the confidentiality of the data to ensure the reliability and authenticity of the questionnaires completed by the participants. All 300 MBA students were willing and able to participate at both time points in the second phase of the formal investigation. 

At Time 1, we collected information regarding sex, age, work experience years, current position years, negative informal information, and individuals’ resistance intention before the organizational change. The total number of usable responses was 255 after eliminating 45 problematic responses. At Time 2 (2 weeks later after Time 1), participants reported resistance intention during the organizational change, perceived sanction, and individual performance. To avoid participants who experienced multiple organizational changes simultaneously from confusing the measurement of organizational-change-related variables at both time points, we recorded the change events and student ID at Time 1 and marked them at Time 2, and finally obtained 215 valid matched questionnaires (the effective response rate was 84.3%).

The sample included 102 men (47.40%) and 113 women (52.60%). The average age of participants was 32.90 years old (SD = 5.86), the average work experience of participants was 10.54 years (SD = 7.05), and the average current position years of participants was 5.21 years (SD = 5.69). Of the respondents, 39.5% experienced management system changes, such as business expansion or combination, organizational structure adjustment, etc.; 38.1% experienced personnel changes, such as personnel transfer, salary adjustment, etc. Participants served a variety of industries such as service, manufacturing, information, and government.

### 3.2. Measures

#### 3.2.1. Informal Information Before Organizational Change (Time 1) 

We adapted three items from Miller et al. [28] to measure this contrast using a five-level semantic difference scale (SDS) [29]. Here, we focused on negative informal information. The interval is expressed by quantifiers with certain magnitudes, with three points indicating the central quantifier (1 = positive, 5 = negative) [30]. A sample item is “Before the organizational change, all kinds of informal information I heard were mostly very negative vs. very positive.” Cronbach’s α was 0.952.

#### 3.2.2. Resistance Intention (Times 1 and 2) 

The resistance intention scale is measured by 3 items adapted from Oreg [23] and Chung et al. [1]. A sample item is “I like to do the same old things rather than try new and different ones”. To investigate resistance intention before the change and during the change, the same three questions were used but the time limit was different. One was the initial stage of hearing the informal information before change and the other was during the implementation period of change. This construct was measured using the 5-point Likert scale from 1 (strongly disagree) to 5 (strongly agree). Cronbach α for Time 1 was is 0.944 and 0.956 for Time 2.

#### 3.2.3. Perceived Sanctions (Time 2)

To measure the perceived sanctions, we used a 3-item scale developed by Furst and Cable [25]. A sample item is “If I do not support the changes that have already been implemented, I may be punished.” Responses to all items ranged from 1 (strongly disagree) to 5 (strongly agree). Cronbach α was 0.809.

#### 3.2.4. Individual Performance (Time 2)

According to the actual situation, employees objectively reported the first performance appraisal after the implementation of change. The appraisal results were A to D (A = 4, D = 1), indicating performance ranging from high to low.

#### 3.2.5. Control Variables (Time 1)

To control for the influence of personal characteristics on employees’ resistance intention to change, we controlled employees’ sex, age, work experience years, and current position years. Sex was dummy coded as 1 = male and 0 = female.

## 4. Data Analysis and Results

### 4.1. Common Method Variance

Except for the objective variable of individual performance, we used nine measurement items of key variables (including negative informal information before the organizational change, resistance intention (Time 1), and perceived sanction) to test the common method variance (CMV) issues at the measurement level. We used the unmeasured latent method construct (ULMC) to test the common method bias of data from the same source using confirmatory factor analysis (CFA) test, in which nested models are compared to formally detect CMV [31,32]. Specifically, the fit of the model with both substantive construct–substantive items and method construct-substantive item loadings can be compared to the fit of a model with only substantive construct–substantive item loadings to determine whether observed relationships can be attributed to both method and substantive variance, thereby indicating the presence of CMV [33]. If the two models are significantly different, evidence exists of method bias. The result showed that the two models are not significantly different (Δχ^2^ = 0.204, Δ*df* = 3, *p* > 0.1) (χ^2^ = Chi Square; *df* = degrees of freedom). Therefore, CMV was acceptable.

### 4.2. Confirmatory Factor Analysis

The construct validity of the research instrument was assessed via confirmatory factor analysis (CFA). To perform a CFA, all the constructs and reflective indicators were depicted and composed as a measurement model in which all constructs were allowed to correlate with each other. We conducted CFA using AMOS 20 (IBM Corp., Armonk, NY, USA) to determine the uniqueness of the study variables. Table 1 shows that the three-factor model fit the data well: *χ*^2^ = 22.492, *df* = 24, comparative fit index (CFI) = 1.000, normed fit index (NFI) = 0.986, goodness of fit index (GFI) = 0.977, adjusted goodness of fit index (AGFI) = 0.957, parsimonious goodness of fit index (PGFI) = 0.521, and root mean square error of approximation (RMSEA) = 0.000. The three-factor model fit the data better than the other alternative models (Table 1) and the fit indices exceeded the acceptable levels suggested in the literature: *χ*^2^/*df* ≤ 5, CFI ≥ 0.9, NFI ≥ 0.9, GFI ≥ 0.9, AGFI ≥ 0.9, PGFI ≥ 0.5, and RMSEA ≤ 0.08 [34,35,36,37,38,39].

### 4.3. Descriptive Data Analysis

Table 2 reports the means, standard deviations, and a correlation matrix showing Pearson’s correlation coefficients for all constructs. We found that negative informal information was significantly positively correlated with resistance intention to change (Time 1) (*r* = 0.363, *p* < 0.05) and resistance intention to change (Time 2) (*r* = 0.437, *p* < 0.01), and resistance intention to change (Time 1) was also found to be highly positive correlated to resistance intention to change (Time 2) (*r* = 0.613, *p* < 0.01. We observed a highly significant negative correlation between resistance intention to change (Time 2) and individual performance (*r* = −0.148, *p* < 0.01). Through an in-depth exploration of related relationships, we laid the foundation for further regression analysis.

### 4.4. Results of Hypothesis Testing

The results from our regression analysis (Table 3) suggested that the negative informal information before organizational change was positively related with resistance intention (Time 1) (*β* = 0.343, *p* < 0.01), and resistance intention (Time 2) was negatively related to individual performance (*β* = −0.106, *p* < 0.05). Thus, Hypotheses 1 and 4 were supported. 

**Figure 2:**
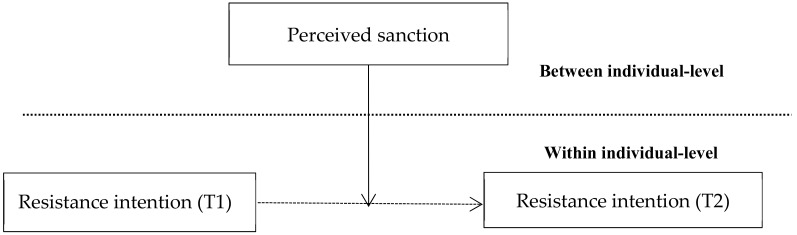
Schematic diagram of a multilevel linear growth model.

To test Hypotheses 2 and 3, a multi-level linear growth model was adopted using HLM software version 6 (Scientific Software International Inc., Lincoln-wood, IL, USA). Applying the multilevel linear growth model can solve two major problems: data nesting with hierarchical structure and repeated measurement or longitudinal research. Consequently, this method helps to compensate for the errors and limitations of the general regression. The data from repeated measurement can also be regarded as nested data with a hierarchical structure [40]. Using a multi-level linear growth model, the trend in individual development (persons within groups) and their own differences (measures within persons) can be analyzed accordingly. The second problem was identified in our research design (Figure 2), therefore, hierarchical linear growth modeling was used to examine the hypotheses. Although a longitudinal study design was adopted, each wave of variables was collected from the same source (except individual performance), which increased the possibility of common method variance [40]. The empirical distinctiveness of variables at Times 1 and 2 was assessed via CFA. The results are presented in Table 1, which supports the hypothesized factor structure for both Time 1 and Time 2, indicating that the current measures possess adequate discriminant validity [32]. Because within-individuals were nested in individuals, we used hierarchical linear growth modeling to examine the effects of perceived sanction on resistance intention development over time [40]. Level 1 represented a within-individual change from Time 1 (T1) to Time 2 (T2), taking time as the independent variable (Table 4, model 2). Level 2 added perceived sanction to the model and individual-level control variables (model 4) and examined whether perceived sanction moderated the effect of resistance intention (T1) on resistance intention (T2). 

As shown in model 2 in Table 4, at the within-individual level, the resistance intention declined significantly with time (*β* = −0.129, *p* < 0.05). Therefore, Hypothesis 2 was supported. Hypothesis 3 posited that resistance intentions weaken faster when more sanctions are perceived. We added the perceived sanction into the individual-level as a cross-level moderator of the time-dependent change in resistance intention. The null model (model 1) in Table 4 shows within-individual variance was 41%, between-individual variance was 60%. Model 2 of Table 4 introduced time (1 = T1 and 2 = T2) as a predictor of within-individual variations in resistance intention (Level 1). Model 4 of Table 4 demonstrates there was no significant cross-level moderating effect of perceived sanction (*β* = 0.108, *p* > 0.05). Therefore, Hypothesis 3 was not supported.

## 5. Discussion

Based on the construal level theory, a framework was developed to identify how negative informal information before organizational change affects employee performance through their resistance intention over time. In general, this study provides a dynamic perspective on the key mechanism of individual resistance intention fading. The main results from this research include: (1) informal negative information before a change is positively related to the resistance intention before the change; (2) resistance intention to organizational change decreases significantly over time, without a cross-level moderation effect of perceived sanction; and (3) resistance intention during the change is negatively related to individual performance. The following subsections highlight the theoretical and practical implications of the findings, as well as the limitations and directions for future research.

### 5.1. Theoretical and Practical Contributions

First, the negative informal information before an organizational change is positively related to the resistance intention before the change. Despite the focus of existing research on the relationship between information communication and employee response when organizational change occurs [41], little is known about how informal information before a change affects resistance intentions. Informal information is often circulated before a change occurs [3], playing the role of pre-announcement of the change, which affects the employees’ cognition and response to the organizational change. The more negative the informal information, the more pressure and discomfort felt by the employees [16], and the more likely their resistance intention is triggered. Therefore, the first key contribution of our research is that we extend the literature on informal communication by offering a conceptual framework to analyze how negative informal information before a change impacts employees’ resistance in the workplace. The findings enhance the current understanding of negative informal information before an organizational change.

Second, resistance intention to the organizational change decreases significantly over time without any impact on the cross-level moderation effect of perceived sanction. According to construal level theory, when employees become aware that the organizational change is approaching and will be inevitable over time, they gradually adapt themselves and begin to rethink and evaluate the situation so that they can be psychologically prepared when the change is implemented [24]. From the initial to the final stages, employees’ perception of and psychology surrounding the change is constantly changing over time. Perceived sanction to the organizational change does not effectively reduce the employees’ resistance intention. This may be related to the employees’ impression of their manager. They may think about sanction as manager dissatisfaction or distrust [25], or perceive managers as “tyrants” who abuse their authority [42]. This perception may further weaken the employee’s sense of liking for managers and encourage them to retaliate by resisting the organizational change [43]. The findings from our research broaden understanding of construal level theory and research by advancing a within-individual perspective of how resistance intention decreases over time. In the literature, employees’ change response appears more as an outcome [25]. Over time, employees’ intention reaction to a change is dynamic, which is worthy of attention [10,40]. Therefore, we applied the time distance of construal level theory to focus on the dynamic process of employees’ resistance intention over time, which also enriches the research on the intention response of employees to organizational change and complements the research on organizational change management. 

Third, the resistance intention during an organizational change is negatively related to individual performance. In other words, the greater the resistance intention of employees during the organizational change, the worse their follow-up performance. If resistance intention is high, the employees hold a disapproving attitude toward the change and they will not actively participate in implementing the change or support the idea of the change, which affects the smooth implementation of the change; thus, their own work performance will be reduced. Employee performance can also objectively reflect the effect of the implementation of the change. Overall, we established a complete framework of the effect of negative informal information on employee performance through resistance intention over time. The findings from this research broaden the construal level theory and research by advancing a within-individual approach and extend the literature on informal information before a change and organizational change management. In addition, we contribute to understanding other downstream outcomes that could be affected by resistance intention to organizational change.

Our findings have practical implications as well. All enterprises inevitably make frequent, continuous, and wide-ranging changes to ensure a sustainable competitive advantage remains on-trend [11]. Organizational change is likely to succeed only if managers continue to reduce employee resistance or seek their support [44], which is why addressing the negative views of employees and promoting their positive input are important for successful implementation of organizational changes. According to our research, employees form a basic affective reaction once they receive the informal information before the change occurs. The informal information may help the employees regain control of the environment and reduce uncertainty. Therefore, informal information before the change can foster the employees’ adjustment in advance and reduce employee resistance during the change. This suggests that informal information is not always harmful or useless; sometimes it helps employees to respond effectively to organizational changes. Our findings emphasize that informal information is universal and inevitable within the organization, but the methods for strategically managing the information have important practical implications. For instance, managers can appropriately disclose some informal information to help employees understand the situation and self-adjust before the organizational change occurs. 

To smoothly implement organizational change, managers often consider sanctions to complete the change more quickly. However, our findings indicate that employee resistance intention will not decline correspondingly with perceived sanctions. Therefore, after the formal implementation of the change, if a manager adopts some soft tactics to allow employees to accept the organizational change, employee resistance may be mitigated, promoting the smooth implementation of the change. 

### 5.2. Limitations and Suggestions for Future Research

Our findings also have several limitations that should be acknowledged. First, using retrospective self-reporting data to measure the effect may have introduced recall effects and hindsight biases [25,45]. For instance, an employee’s review of an event may not fully reflect the reaction of the change at the time. However, if we can collect the data from the companies undergoing the change and track the data during the change, the measurements will be more accurate and efficient. Therefore, in future research, we could continue to explore more appropriate and rigorous methods for data collection to reduce bias and obtain more accurate results.

Another limitation is that we only examined the perceived sanction as a moderator of the reduction effect of resistance intention to the organizational change over time, but it is possible that other variables with respect to change information may moderate this effect. For example, how employees react to organizational change at different frequencies of informal information could be examined. Resistance intention may not wane if the frequency of negative informal information increases. Resistance is the most common response to change when employees receive negative information, but employees may choose other responses, such as psychological withdrawal or interaction avoidance. Employees may choose different responses depending on their personality traits or conformity pressures. Consequently, future research could investigate the relationship between personality traits or conformity pressures and employee reactions over time. This research would enrich the literature on organizational change communication and information management.

Despite the strength of our design across multiple time points and sources, we cannot completely rule out alternative causal interpretations. Future research may benefit from improved experimental design and multiple samples to reduce the causal relationship concerns. The sample of our study was limited to a central province in China. Despite the sample of MBA students being distributed in various positions and had experienced a variety of change events, the external validity of the sample was guaranteed to some extent. Due to the different levels of economic and cultural development in various regions of China, the results of a similar study in a different region could differ. For example, in economically developed regions, employees may be more likely to accept changes. Therefore, the universality of conclusions needs to be further explored. In future research, a wider range of samples from different regions needs to be collected to improve the representativeness and robustness.

## 6. Conclusions

Although effective change management is a key organizational capability, in many situations, most of the implemented changes do not meet the expected goals. Successful change implementation requires effective communication and management to reduce employees’ resistance to change. Informal information is important to both organizations and individuals; however, a limited range of research has focused on informal information before an organizational change. Little is known about the dynamic changes in the intention reaction of employees over time. However, understanding the micro process of employees’ adjustment is crucial because organizational change is only likely to succeed if managers continue to reduce the employees’ resistance or seek their support. 

In this study, we aimed to reveal the relationship between negative informal information and individual performance through investigation of the micro process of within-person dynamics over time. Based on construal level theory, we examined how negative informal information is related to employees’ work performance and the change in resistance intention over time. As expected, resistance intention (Time 1) predicted employees’ intention response during the change and their performance at work. The employees who intend to resist are more upset about the change and find it harder to work effectively. Even though individuals’ resistance intention waned over time, individual performance was still negatively impacted, which cannot be ignored. 

The findings from this research contribute to knowledge by (1) extending the literature on informal communication by offering a conceptual framework to analyze how negative informal information before a change impacts employee resistance intention in the workplace, (2) enhancing the current understanding of negative informal information before change occurrence, and (3) elucidating the dynamics of personal resistance intention over time.

## Figures and Tables

**Table 1:** Results of confirmatory factor analysis (CFA) of variables.

MODEL	χ^2^	*df*	χ^2^/*df*	CFI	NFI	GFI	AGFI	PGFI	RMSEA
One-factor model	875.564	27	32.428	0.459	0.455	0.573	0.288	0.344	0.383
Two-factor model ^a^	225.385	26	8.669	0.873	0.860	0.805	0.662	0.465	0.189
Three-factor model	22.492	24	0.937	1.000	0.986	0.977	0.957	0.521	0.000

^a^ Combining resistance intention (time 1) and perceived sanction.

**Table 2:** Descriptive statistics and correlation matrix for all constructs.

Variable	M	SD	1	2	3	4	5	6	7
1 Age	32.90	5.86							
2 Work experience years	10.54	7.05	0.956 **						
3 Current position years	5.21	5.69	0.556 **	0.610 **					
4 Negative Informal information	2.87	1.10	−0.119	−0.128	−0.012				
5 Resistance intention (T1)	2.71	1.05	0.021	0.025	0.105	0.363 *			
6 Resistance intention (T2)	2.58	0.99	−0.142 *	−0.143 *	−0.061	0.437 **	0.613 **		
7 Perceived sanction	3.29	0.83	0.128	0.125	0.017	0.076	0.114	0.213	
8 Individual performance	3.04	0.71	−0.008	0.009	0.001	−0.053	−0.048	−0.148 *	−0.107

Note: M, mean; *N* = 215; * *p* < 0.05, ** *p* < 0.01.

**Table 3:** Regression analysis results.

Independent Variable	Dependent Variable			
	Resistance Intention (Time 1)		Individual Performance	
	Model 1	Model 2	Model 3	Model 4
Constant	2.322 *	1.403	3.734	4.045
Sex	0.264	0.228	−0.104	−0.095
Age	0.008	0.004	−0.026	−0.027
Work experience years	−0.014	0.000	0.022	0.020
Current position years	0.025	0.018	−0.002	−0.001
Negative informal information		0.343 **		
Resistance intention (Time 2)				−0.106 *
*R* ^2^	0.029	0.155	0.009	0.030

Note: *N* = 215, * *p* < 0.05, ** *p* < 0.01.

**Table 4:** Hierarchical linear models predicting the decrease of resistance intention from T1 to T2.

Variable	Resistance Intention			
	Model 1	Model 2	Model 3	Model 4
Control variables				
Sex	0.215	0.215	0.215	0.215
Age	−0.001	−0.001	−0.001	−0.001
Work experience years	−0.019	−0.019	−0.019	−0.019
Current position years	0.019	0.019	0.019	0.019
Main effect within-individual level (level 1)				
Time		−0.129 *		−0.129 *
Cross-level effect (from level 2 to level 1)				
Perceived sanction			0.232 **	0.108
σ^2^ (Within-individual)	0.410	0.403	0.410	0.401
τ (Between-individual)	0.600 *	0.603	0.600	0.604

Note: *N* = 215; * *p* < 0.05, ** *p* < 0.01.

## References

[1] Chung G.H., Choi J.N., Du J. (2017). Tired of innovations? Learned helplessness and fatigue in the context of continuous streams of innovation implementation. J. Organ. Behav..

[2] Petrou P., Demerouti E., Schaufeli W.B. (2018). Crafting the change: The role of employee job crafting behaviors for successful organizational change. J. Manag..

[3] Herzig S.E., Jimmieson N.L. (2006). Middle managers’ uncertainty management during organizational change. Leadersh. Organ. Dev. J..

[4] Cameron E., Green M. (2020). Making Sense of Change Management: A Complete Guide to the Models, Tools and Techniques of Organizational Change.

[5] Al-Haddad S., Kotnour T. (2015). Integrating the organizational change literature: A model for successful change. J. Organ. Chang. Manag..

[6] Smet K., Vander Elst T., Griep Y., De Witte H. (2016). The explanatory role of rumours in the reciprocal relationship between organizational change communication and job insecurity: A within-person approach. Eur. J. Work Organ. Psychol..

[7] Elving W.J.L. (2005). The role of communication in organisational change. Corp. Commun. An Int. J..

[8] DiFonzo N., Bordia P. (2007). Rumor, gossip and urban legends. Diogenes.

[9] Beersma B., Van Kleef G.A. (2011). How the Grapevine Keeps You in Line: Gossip Increases Contributions to the Group. Soc. Psychol. Personal. Sci..

[10] Matusik J.G., Hollenbeck J., Matta F.K., Oh J.K. (2019). Dynamic Systems Theory and Dual Change Score Models: Seeing Teams through the Lens of Developmental Psychology. Acad. Manag. J..

[11] Cheng C., Lau H.-P.B., Chan M.-P.S. (2014). Coping flexibility and psychological adjustment to stressful life changes: A meta-analytic review. Psychol. Bull..

[12] Voelkle M.C., Brose A., Schmiedek F., Lindenberger U. (2014). Toward a Unified Framework for the Study of Between-Person and Within-Person Structures: Building a Bridge Between Two Research Paradigms. Multivar. Behav. Res..

[13] Grosser T.J., Lopez-Kidwell V., Labianca G., Ellwardt L. (2012). Hearing it through the grapevine: Positive and negative workplace gossip. Organ. Dyn..

[14] Bordia P., Kiazad K., Restubog S.L.D., DiFonzo N., Stenson N., Tang R.L. (2014). Rumor as Revenge in the Workplace. Group Organ. Manag..

[15] Brady D.L., Brown D.J., Liang L.H. (2017). Moving beyond assumptions of deviance: The reconceptualization and measurement of workplace gossip. J. Appl. Psychol..

[16] Bordia P., Jones E., Gallois C., Callan V.J., Difonzo N. (2006). Management are aliens! Rumors and stress during organizational change. Group Organ. Manag..

[17] Barmeyer C., Mayrhofer U., Wurfl K. (2019). Informal information flows in organizations: The role of the Italian coffee break. Int. Bus. Rev..

[18] Rosnow R.L. (1991). Inside rumor: A personal journey. Am. Psychol..

[19] Beersma B., Van Kleef G.A. (2012). Why people gossip: An empirical analysis of social motives, antecedents, and consequences. J. Appl. Soc. Psychol..

[20] Johlke M.C., Duhan D.F. (2000). Supervisor communication practices and service employee job outcomes. J Serv. Res..

[21] Hargie T., Tourish D. (2000). Handbook of Communication Audits for Organisations.

[22] Vander Elst T., Sverke M., De Witte H., Näswall K., Hellgren J., Baillien E., De Cuyper N., De Witte H. (2010). The role of organizational communication and participation in reducing job insecurity and its negative association with work-related well-being. Econ. Ind. Democr..

[23] Oreg S. (2003). Resistance to change: Developing an individual differences measure. J. Appl. Psychol..

[24] Liu Y., Perrewe P.L. (2005). Another look at the role of emotion in the organizational change: A process model. Hum. Resour. Manag. Rev..

[25] Furst S.A., Cable D.M. (2008). Employee resistance to organizational change: Managerial influence tactics and leader-member exchange. J. Appl. Psychol..

[26] Higgins C.A., Judge T.A., Ferris G.R. (2003). Influence tactics and work outcomes: A meta-analysis. J. Organ. Behav..

[27] Aaker J., Drolet A., Griffin D. (2008). Recalling mixed emotions. J. Consum. Res..

[28] Miller V.D., Johnson J.R., Grau J. (1994). Antecedents to willingness to participate in a planned organizational change. J. Appl. Commun. Res..

[29] Osgood C.E., Suci G.J., Tannenbaum P.H. (1957). The Measurement of Meaning.

[30] Lopes J.D.L., Nogueira-Martins L.A., Andrade A.L.D., Barros A.L.B.L.D. (2011). Semantic differential scale for assessing perceptions of hospitalized patients about bathing. Acta Paul. Enferm..

[31] Williams L.J., Cote J.A., Buckley M.R. (1989). Lack of method variance in self-reported affect and perceptions at work: Reality or artifact?. J. Appl. Psychol..

[32] Podsakoff P.M., MacKenzie S.B., Lee J.-Y., Podsakoff N.P. (2003). Common method biases in behavioral research: A critical review of the literature and recommended remedies. J. Appl. Psychol..

[33] Richardson H.A., Simmering M.J., Sturman M.C. (2009). A tale of three perspectives: Examining post hoc statistical techniques for detection and correction of common method variance. Organ. Res. Methods.

[34] Bentler P.M., Bonett D.G. (1980). Significance tests and goodness of fit in the analysis of covariance structures. Psychol. Bull..

[35] Bentler P.M. (1990). Comparative fit indexes in structural models. Psychol. Bull..

[36] Doll W.J., Xia W., Torkzadeh G. (1994). A confirmatory factor analysis of the end-user computing satisfaction instrument. MIS Q..

[37] Henry J.W., Stone R.W. (1994). A structural equation model of end-user satisfaction with a computer-based medical information system. Inf. Resour. Manag. J..

[38] Hu L.T., Bentler P.M. (1999). Cutoff criteria for fit indexes in covariance structure analysis: Conventional criteria versus new alternatives. Struct. Equ. Model. Multidiscip. J..

[39] Loo R., Thorpe K. (2000). Confirmatory factor analyses of the full and short versions of the Marlowe-Crowne Social Desirability Scale. J. Soc. Psychol..

[40] Du J., Shin Y., Choi J.N. (2015). Convergent perceptions of organizational efficacy among team members and positive work outcomes in organizational teams. J. Occup. Organ. Psychol..

[41] Oreg S. (2006). Personality, context, and resistance to organizational change. Eur. J. Work Organ. Psychol..

[42] Ashforth B. (1994). Petty tyranny in organizations. Hum. Relat..

[43] Tepper B.J. (2000). Consequences of abusive supervision. Acad. Manag. J..

[44] Van Den Heuvel M., Demerouti E., Bakker A.B., Schaufeli W.B. (2013). Adapting to change: The value of change information and meaning-making. J. Vocat. Behav..

[45] Pohl R.F., Hell W. (1996). No reduction in hindsight bias after complete information and repeated testing. Organ. Behav. Hum. Decis. Process..

